# Photochemical Production and Behavior of Hydroperoxyacids in Heterotrophic Bacteria Attached to Senescent Phytoplanktonic Cells

**DOI:** 10.3390/ijms140611795

**Published:** 2013-06-03

**Authors:** Morgan Petit, Richard Sempéré, Frédéric Vaultier, Jean-François Rontani

**Affiliations:** 1 Mediterranean Institute of Oceanography (MIO), Aix Marseille Université, CNRS/INSU, IRD, UM 110, 13288 Marseille, France; E-Mails: richard.sempere@univ-amu.fr (R.S.); frederic.vaultier@univ-amu.fr (F.V.); jean-francois.rontani@univ-amu.fr (J.-F.R.); 2 Mediterranean Institute of Oceanography (MIO), Université de Toulon, CNRS/INSU, IRD, UM 110, 83957 La Garde, France

**Keywords:** phytoplankton, photodegradation, attached heterotrophic bacteria, *cis*-vaccenic acid, singlet oxygen transfer

## Abstract

The photooxidation of cellular monounsaturated fatty acids was investigated in senescent phytoplanktonic cells (*Emiliania huxleyi*) and in their attached bacteria under laboratory controlled conditions. Our results indicated that UV-visible irradiation of phytodetritus induced the photooxidation of oleic (produced by phytoplankton and bacteria) and *cis*-vaccenic (specifically produced by bacteria) acids. These experiments confirmed the involvement of a substantial singlet oxygen transfer from senescent phytoplanktonic cells to attached bacteria, and revealed a significant correlation between the concentration of chlorophyll, a photosensitizer, in the phytodetritus and the photodegradation state of bacteria. Hydroperoxyacids (fatty acid photoproducts) appeared to be quickly degraded to ketoacids and hydroxyacids in bacteria and in phytoplanktonic cells. This degradation involves homolytic cleavage (most likely induced by UV and/or transition metal ions) and peroxygenase activity (yielding epoxy acids).

## 1. Introduction

It is generally accepted that the majority of the marine particulate organic matter (POM) produced by phytoplankton (phytodetritus), in the oceanic euphotic layer, is recycled within the food web and microbial loop in surface water [1], and that only a small fraction of POM is exported by particles, sinking towards the deep ocean and seafloor [2]. POM may be considered as a critical intermediary between primary production and sediment preservation [3,4]. It is well known that, in the water column, phytodetritus could be decomposed by: (i) zooplankton grazing, and (ii) enzymatic degradation by attached and free living heterotrophic bacteria [5–8]. However, there is a lack of data concerning the photooxidation and autoxidation of these particles, although the importance of these processes in the marine environment has been previously demonstrated [9,10].

During the senescence of phototrophic organisms, visible light and UV-induced photosensitized degradation processes intensively act on numerous cellular components [11,12] due to the presence of a very efficient photosensitizer, chlorophyll [13,14]. When a chlorophyll molecule absorbs a quantum of light energy, an excited singlet state (^1^Chl) is formed. In healthy cells, the absorbed energy is primarily used during photosynthetic reactions [13], whereas a small proportion (less than 0.1%) of ^1^Chl may undergo intersystem crossing to form the longer lived triplet state (^3^Chl) [14]. ^3^Chl is not only potentially damaging by itself in type I reactions [14] but it may also generate toxic oxygen species, such as singlet oxygen (^1^O_2_) and, to a lesser extent, the superoxide anion (O_2_·^−^ ) by reaction with ground state oxygen (^3^O_2_) (type II photoprocesses). Oxidative damage to cells is usually limited by many photoprotective compounds (e.g., carotenoids, tocopherols, and ascorbic acid) and enzymes (e.g., superoxide dismutase) in chloroplasts [13,15]. In contrast, in senescent phototrophic organisms (in which photosynthetic reactions are not functional), an accelerated rate of ^3^Chl formation and toxic oxygen species, exceeding the quenching capacity of the photoprotective system, has been reported [16], which leads to the photodegradation of cellular components (photodynamic effect) [17]. Moreover, note that the lifetime of ^1^O_2_ produced from sensitizers in a lipid-rich hydrophobic environment, such as a cellular medium, could be longer, and its potential diffusive distance is greater, than in an aqueous solution [18]. Therefore, it is not surprising that photodegradation processes act on the majority of unsaturated lipid components of senescent phytoplankton, including sterols, unsaturated fatty acids, chlorophyll phytyl side chain, carotenoids, and alkenes (for reviews, see [11,19]).

The effects of photooxidation, however, are not limited to chloroplasts. Indeed, during the senescence of higher plants ^1^O_2_ can migrate outside the chloroplasts and chemically react with unsaturated components of cuticular waxes [20]. In the case of senescent phytoplanktonic cells, ^1^O_2_ can induce the degradation of the heterotrophic bacteria attached to particles [21]. Indeed, phytodetritus constitute hydrophobic microenvironments in which the lifetime and potential diffusive distance of ^1^O_2_ may be sufficiently long to allow its transfer to attached bacteria. Interestingly, the photooxidation of *cis*-vaccenic acid (a fatty acid typical of Gram negative bacteria [22,23]) has been observed after solar irradiation of non-axenic phytodetritus, whereas this compound appeared to be unaffected after the irradiation of isolated bacteria [24], which indicates an external origin for the oxidant species. Cellular damages resulting from the transfer of substantial amounts of ^1^O_2_ from phytoplanktonic cells to their attached bacteria may be significant due to the lack of efficient photoprotective and antioxidant systems in these microorganisms [25], and can affect their capability in degrading particulate organic matter (POM). Such a transfer of ^1^O_2_, towards bacteria has also been observed *in situ* in several particulate organic matter samples [9,26–28], and may help to explain the relative recalcitrance of strongly abiotically degraded suspended POM towards biotic degradation [9].

The deleterious effects of ^1^O_2_ towards bacteria are well-known. Indeed, the photodynamic killing of bacteria using light, in combination with ^1^O_2_-producing photosensitizers to induce a phototoxic reaction, has been intensively studied in the literature [22,26,27]. Various classes of chemical compounds, including phenothiazines, phthalocyanines, and porphyrins, which have photoactive properties, have been successfully tested as photo-inactivating agents against Gram-positive and Gram-negative bacteria [29–32]. The present study focuses on the effects of a natural source of ^1^O_2_ (senescent phytoplanktonic cells) to their attached bacteria. These processes, which have been ignored in the literature until now, might strongly limit bacterial growth in the seawater column and, consequently, contribute to a better preservation of algal material toward biotic degradation during settling [33].

In this study, we intend to (i) confirm the role played by ^1^O_2_ in altering the bacteria attached to phytodetritus, (ii) correlate the photodegradation state of the bacteria with the evolution of the concentration of chlorophyll (sensitizer) in the phytodetritus, and (iii) compare the behavior of photochemically-produced hydroperoxides in bacteria and phytoplankton cells under PAR + UV irradiations. For this purpose, the photooxidation of oleic (produced by phytoplankton and bacteria) and *cis*-vaccenic (specifically produced by bacteria) acids in a non-axenic senescent culture of *Emiliania huxleyi* was investigated under laboratory controlled conditions.

## 2. Results and Discussion

### 2.1. ^1^O_2_ Transfer

**Figure 1:**
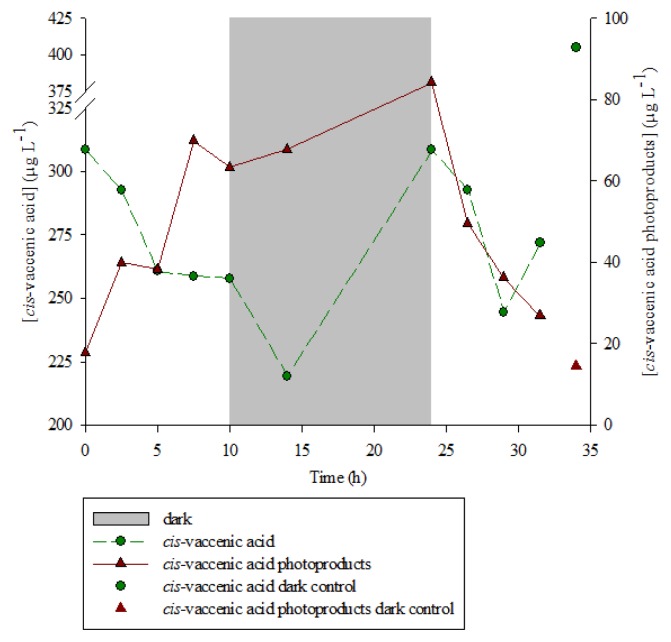
*Cis*-vaccenic acid and *cis*-vaccenic acid photoproducts concentration during the photodegradation of non-axenic *E. huxleyi* strain TW1 with a solar simulator.

**Figure 2:**
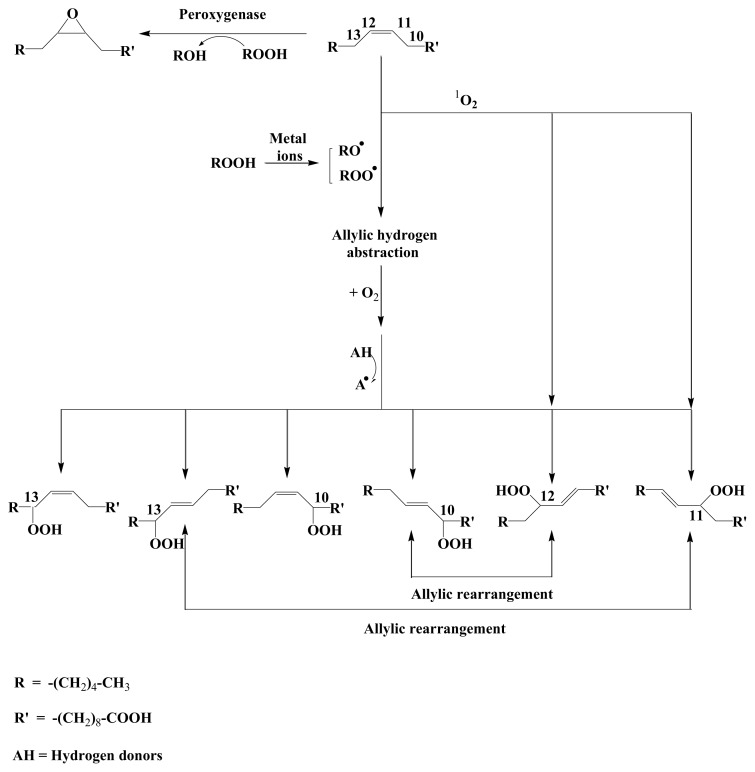
Hydroperoxides from autoxidation and ^1^O_2_-mediated photoprocesses of octadec-11-enoic acid.

**Figure 3:**
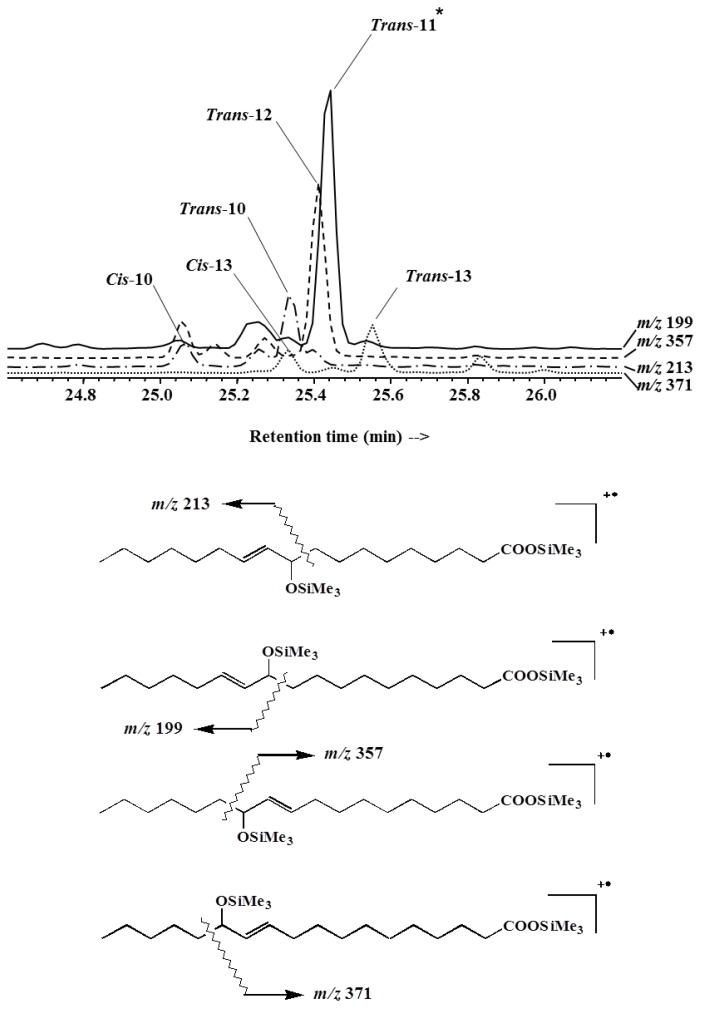
Mass chromatograms of *m/z* 199, 357, 213, and 371, revealing the presence of hydroxyacids derived from octadec-11-enoic acid. The asterisk (*) indicates that the numbers refer to the oxygenated carbon atoms and not to the olefinic centers whose configuration is indicated.

The results (Figure 1) revealed that during the first 10 h of light exposure, the concentration of *cis*-vaccenic acid (bacterial tracer) decreased from 309 to 258 μg·L^−1^ (linear regression, *n* = 5, *r*^2^ = 0.83, *p*-value < 0.05, slope −5.4) while the concentration of the products from the oxidation of *cis*-vaccenic acid (hydroperoxides, hydroxyacids and ketoacids) increased from 18 to 64 μg·L^−1^ (linear regression, *n* = 5, *r*^2^ = 0.83, *p*-value < 0.05, slope 4.8). Furthermore, the sum of the *cis*-vaccenic acid concentration and the oxidation products concentration stayed approximately constant (321.6 ± 13.3 μg·L^−1^) during the first 10 h (Table 1), showing that this degradation could be considered as a conservative reaction. Due to experimental requirement (size of the solar simulator limiting the number of samples), we were unable to do replicate, so the reproducibility of this experiment was tested on three different strains of *E. huxleyi* (TW1 from the Roscoff marine station, CS814 and CS57 from the CSIRO) with the same experimental conditions. Assuming a first order reaction, reaction rate constants (*k*) of photoproducts formation were calculated during the light period (Table 2) and showed an average *k* value of 0.153 ± 0.018 h^−1^. Singlet oxygen-mediated photooxidation of monounsaturated fatty acids leads to the formation of hydroperoxides at each carbon of the original double bond [34]. Thus, photooxidation of vaccenic acid produces a mixture of 11- and 12-hydroperoxides with an allylic *trans*-double bond [35], affording 13-*trans* and 10-*trans* hydroperoxides respectively, after stereoselective radical allylic rearrangement [36]. In contrast, free radical oxidation processes primarily involve allylic hydrogen abstraction processes and yield mixtures of six *cis* and *trans* isomeric allylic hydroperoxides [35,36]. Therefore, based on the dominance of 11-hydroxyoctadec-*trans*-12-enoic and 12-hydroxyoctadec-*trans*-10-enoic acids among the *cis*-vaccenic degradation products (photooxidation and autoxidation products) observed in our experiment (Figures 2 and 3), its degradation was mainly attributed to ^1^O_2_ transfer from the phytodetritus to the attached bacteria during the exposure to light [21]. As the ^1^O_2_ lifetime is more important in D_2_O than in H_2_O (65 and 3.1 μs, respectively) [37], the increase of *cis*-vaccenic acid oxidation products (ranging from 2.5% to 76.5% relative to the experiment carried out in pure H_2_O) observed when the D_2_O concentration increased from 25% to 100% (Table 3) reinforces the idea of the involvement of ^1^O_2_ during *cis*-vaccenic oxidation. The nonlinear increase of the percentage of *cis-*vaccenic photooxidation products with the percentage of D_2_O observed (Table 3) was attributed to the involvement of competitive heterolytic cleavage of hydroperoxides induced by the augmentation of pH [38]. Indeed, the pH of D_2_O (7.43 at 25 °C) is higher than this H_2_O (6.99 at 25 °C). Moreover, it is very important to note that we failed to detect *cis*-vaccenic photoproducts after irradiation of the isolated bacterial community for 7.5 h (Table 4).

**Figure 4:**
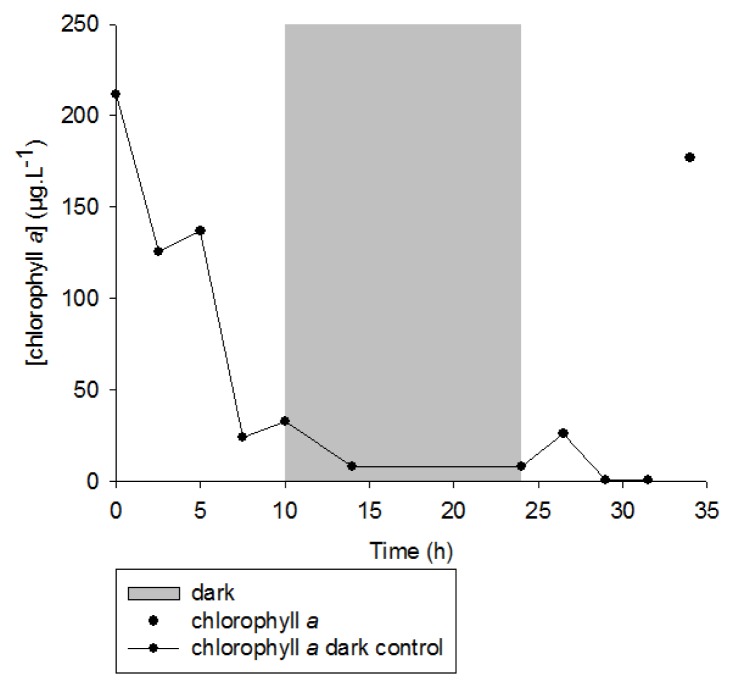
Concentration of chlorophyll *a* during the photodegradation of non-axenic *E. huxleyi* cells with a solar simulator.

**Figure 5:**
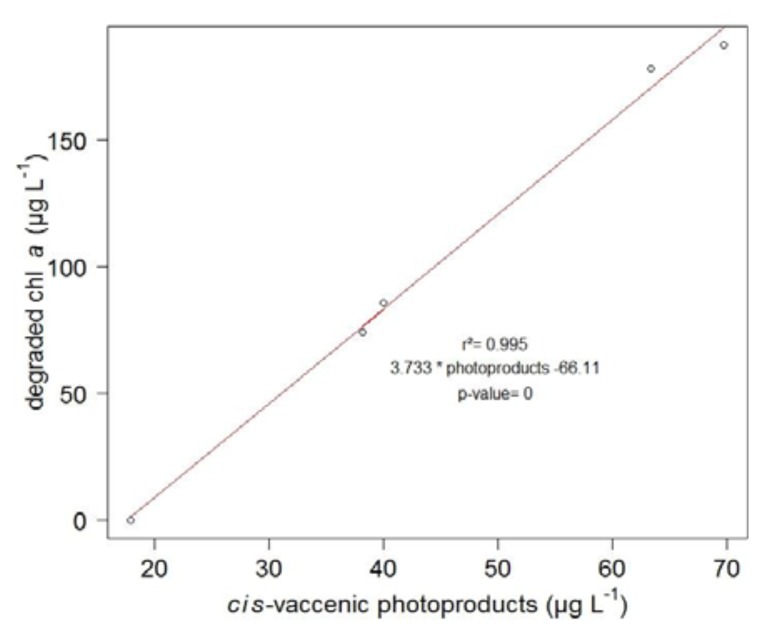
Plot of the correlation between the amount of degraded chlorophyll *a* and produced *cis*-vaccenic acid photoproducts during the photodegradation (first light period, five hours) of non-axenic *E. huxleyi* cells with a solar simulator.

Similarly, during the irradiation period, the concentration of chlorophyll *a* in the senescent cells of *E. huxleyi* strongly decreased (from 210 to 33 μg·L^−1^) (Figure 4). The highly significant (*n* = 5, *r*^2^ = 0.99, *p*-value < 0.001) correlation observed (Figure 5) between the amounts of degraded chlorophyll and produced *cis*-vaccenic acid photoproducts, and the lack of *cis*-vaccenic photoproducts in the bacterial control strongly suggest that the photooxidation of *cis*-vaccenic acid in bacteria is associated with the photosensitizing properties of chlorophyll *a* in senescent *E. huxleyi* cells.

During the dark period (from 10 to 24 h), the photosensitized degradation processes (^1^O_2_ transfer) logically decreased (Figure 1), the formation of *cis*-vaccenic acid oxidation photoproducts slightly increased (from 63.4 to 84.2 μg·L^−1^), this is most likely due to the involvement of autoxidation (free radical-mediated oxidation) processes. Indeed, the reaction of ^1^O_2_ with unsaturated components of the outer lipopolysaccharide membrane of Gram-negative bacteria leads to the formation of reactive secondary products, such as peroxyl radicals, which may subsequently accentuate cell damages [39]. This assumption is well supported by the slight increase (not-shown) in the proportion of 13-hydroxyoctadec-*cis*-11-enoic and 10-hydroxyoctadec-*cis*-11-enoic acids (which are specific tracers of autoxidative processes [26]) observed during the incubation in darkness. The important increase of the *cis*-vaccenic acid (bacterial marker) concentration, from 220 to 308 μg·L^−1^ between 15 and 20 h, was attributed to the bacterial growth of free and attached bacteria. A similar result was observed in the dark control where the concentration reached 404 μg·L^−1^ after 34 h of incubation. It may be noted that the photooxidation of senescent phytoplankton cells during the light period should induce the release of dissolved organic carbon, thus permitting an effective growth of bacterial community during the dark period. Those results confirmed that, during irradiation, bacterial growth seems to be limited by the photooxidation process and in a less proportion way by the autoxidation process.

**Figure 6:**
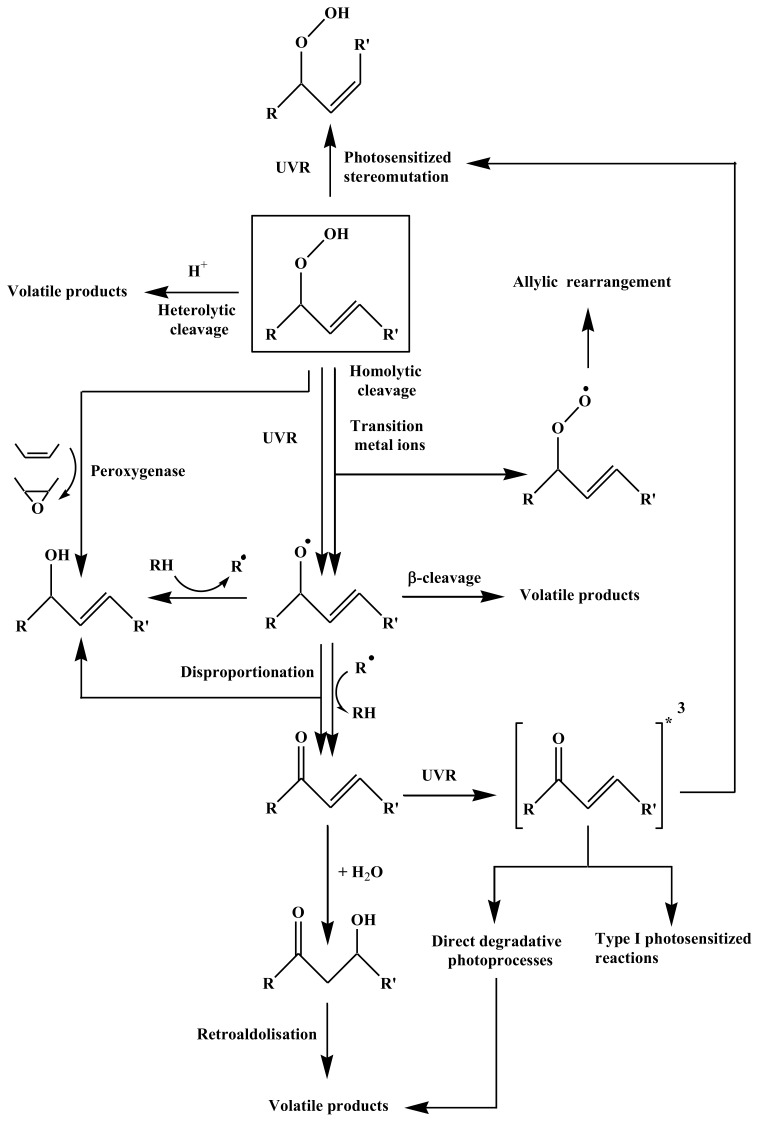
Proposed pathways for the degradation of allylic hydroperoxyacids in senescent phytoplanktonic cells and attached heterotrophic bacteria (modified from [12]).

Interestingly, exposure to light after a dark period induced a strong decrease of *cis*-vaccenic acid (Figure 1), this degradation was attributed to the slight increase of chlorophyll *a* concentration (resulting from a slight phytoplanktonic growth) observed after 25 h of experiment (Figure 4), allowing production of ^1^O_2_ and, thus, photooxidation of the *cis*-vaccenic acid. The faster degradation rate observed during the second light period is probably due to bacterial cell damage, induced during the first light period, favouring the migration of ^1^O_2_ in membranes. The increase of *cis*-vaccenic acid observed, between 28 and 32 h (*i.e.*, when chlorophyll has totally disappeared) (Figure 4), confirms that the degradation of this acid is linked to the involvement of chlorophyll-induced type II photoprocesses. At the return to light conditions, we also noted a substantial decrease in the concentration of the *cis*-vaccenic acid photoproducts (Figure 1). While during the first light period, the formation of *cis*-vaccenic photoproducts by singlet oxygen was faster than their degradation by UV or metal ions (Figure 6), during the second light period, due to the strong degradation of the photosensitizer, the formation of *cis*-vaccenic photoproducts slows down and degradation processes become dominant. Indeed, hydroperoxides absorb in the UVR range [40], and redox-active metal ions may play a very important role in the homolysis of these compounds because they are ubiquitous, active in many forms, and trace quantities are sufficient for effective catalysis [41]. Only metals undergoing one-electron transfers appear to be active catalysts; these metals include cobalt, iron, copper, manganese, magnesium, and vanadium.

9,10- and 11,12-epoxyoctadecanoic acids (concentrations ranging from 0.7 to 2.1 μg·L^−1^) could be detected during all the experiment. Such compounds can be formed by the adding of a peroxyl radical to a double bond [42], followed by a fast intramolecular homolytic substitution [43]. In the case of monounsaturated fatty acids (such as *cis*-vaccenic acid), such a formation is very unlikely. Indeed, this addition only becomes competitive (relative to the abstraction of an allylic hydrogen atom) in the case of conjugated, terminal, or tri-substituted double bonds [41]. The formation of these epoxy acids was attributed to the involvement of peroxygenases (hydroperoxide-dependent oxygenases) during the abiotic degradation of phytoplankton and the attached bacteria. These enzymes, which play a protective role against the deleterious effects of hydroperoxides *in vivo* [44], catalyze the epoxidation of unsaturated fatty acids in the presence of alkyl hydroperoxides as co-substrates (Figure 2).

### 2.2. Degradation of Hydroperoxides

An additional photodegradation experiment was conducted to investigate how hydroperoxides are degraded into hydroxyl acids and ketoacids within bacteria and phytoplanktonic cells, respectively.

**Figure 7:**
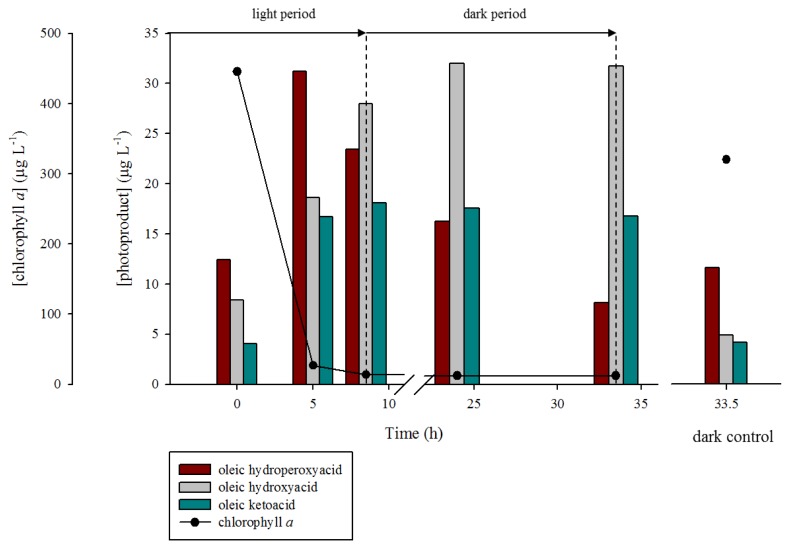
Concentrations of oleic acid degradation photoproducts and chlorophyll *a* during the photodegradation of non-axenic *E. huxleyi* strain TW1 with a solar simulator.

**Figure 8:**
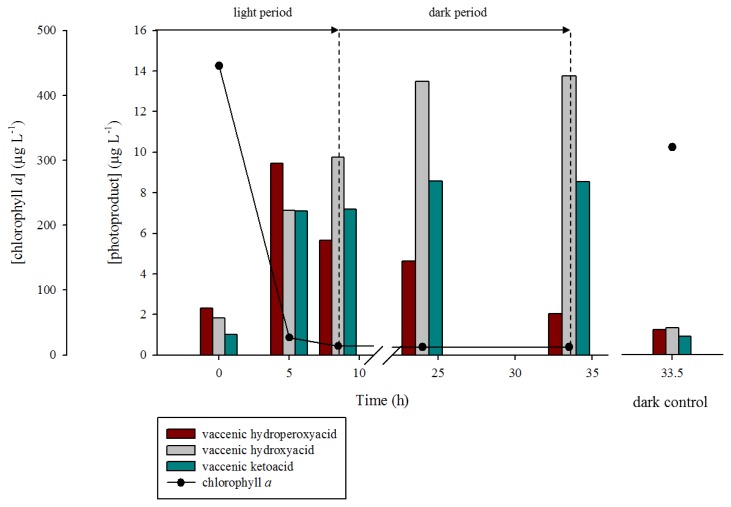
Concentrations of *cis*-vaccenic acid degradation photoproducts and chlorophyll *a* during the photodegradation of non-axenic *E. huxleyi* strain TW1 with a solar simulator.

During the first five hours of the illumination period, a strong production of oleic and vaccenic hydroperoxyacids was observed (from 12.4 to 31.2 μg·L^−1^ and 2.3 to 9.4 μg·L^−1^, respectively) (Figures 7 and 8). During this period, the corresponding hydroxyacids and ketoacids were produced. This production was attributed to the homolytic cleavage of hydroperoxides (most likely induced by UV irradiation [40]). Indeed, as summarized in Figure 6, allylic hydroperoxides may undergo (i) reduction by peroxygenase activity [44]; (ii) heterolytic cleavage catalyzed by protons [34]; (iii) homolytic cleavage induced by transition metal ions [45,46] or UVR [40]; and (v) photosensitized *cis-trans* isomerization [12]. Homolytic cleavage of hydroperoxides (catalyzed by metal ions) leads to the formation of allylic peroxyl or alkoxyl radicals. The resulting alkoxyl radicals can subsequently (i) lead to the formation of volatile products after β-cleavage; (ii) lose a hydrogen atom to produce ketoacids, or (iii) react with another molecule and abstract a hydrogen atom to yield hydroxyacids. Note that hydroxyacids and ketoacids may also result from the disproportionation of two alkoxyl radicals. Allylic ketoacids resulting from the degradation of hydroperoxides may be excited to a triplet state by absorption in the UVR range. This triplet state can then (i) induce type I photosensitized reactions; (ii) lead to volatile products by direct photodegradative processes; or (iii) induce *cis-trans* stereomutation of double bonds [40,47]. Allylic keto groups can also react with water to form β-ketols, which can subsequently be cleaved to volatile compounds by retroaldolisation (Figure 6).

After five hours of irradiation, the concentrations of hydroperoxyacids decreased due to the observed decrease of ^1^O_2_ transfer resulting from the strong degradation of chlorophyll (sensitizer). During the dark period (from 8.5 to 33.5 h), the production of oleic and vaccenic hydroperoxyacids logically stopped and their concentration decreased by 74% and 79% for oleic and vaccenic acids, respectively. This degradation can be attributed to: (i) homolytic cleavage catalyzed by transition metal ions [45,46] (resulting in the formation of hydroxyacids and ketoacids) and (ii) reduction by peroxygenase activity [44] (resulting in the formation of hydroxyacids and epoxyacids) (Figure 6). The fact that the concentration of the oleic and vaccenic ketoacids remained practically constant during the dark period, whereas the concentrations of hydroxyacids continued to increase strongly, suggests the additional involvement of peroxygenase [44] or hydroperoxide reductase [48]. These two types of enzymes catalyze the reduction of hydroperoxides to their corresponding alcohols. Although the involvement of hydroperoxide reductase (using NADPH to reduce hydroperoxides) cannot be totally excluded, we favor the involvement of peroxygenases based on the strong increase (120%) of the total epoxyacids concentration (*i.e.*, oleic + vaccenic epoxyacids) observed during the incubation period.

These results demonstrate that oleic and vaccenic hydroperoxyacids are rapidly degraded in phytodetritus and bacteria. This degradation primarily yields hydroxyacids and ketoacids during irradiation and hydroxyacids under darkness. Assuming first order kinetic no difference was observed between the reaction rate constant (*k*) of oleic hydroperoxyacid degradation (*k* = 0.042 h^−1^) and vaccenic hydroperoxyacid degradation (*k* = 0.045 h^−1^).

## 3. Experimental Section

### 3.1. Algal and Bacterial Material Production

For each experiment, *E. huxleyi* strain TW1 from the Roscoff marine station culture collection was grown in 500 mL of *f*/2 medium under non-axenic conditions at 17 °C, in a constant environmental-controlled cabinet under an irradiance of 36 W·m^−2^ (Osram, Fluora, 12:12 h light:dark cycle), until a stationary phase was obtained.

A bacterial community previously isolated from a culture of *E. huxleyi* was grown in a mixture of synthetic seawater, pyruvate, and acetate at 20 °C.

### 3.2. Photodegradation Experiments

**Figure 9:**
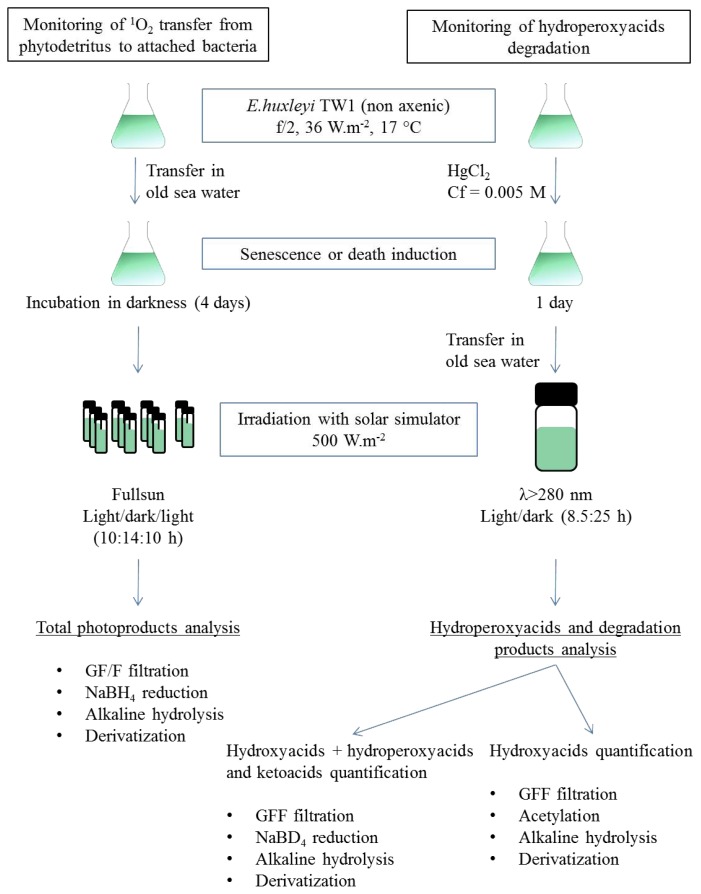
Singlet oxygen transfer and hydroperoxyacid degradation experiments.

*E. huxleyi* cells, in the stationary phase, were transferred (after centrifugation, 3500 rpm for 5 min) into 500 mL of old natural seawater and incubated under darkness, for 4 days, to induce senescence. The ease of bleaching of the resulting cells during the subsequent irradiation attested to their senescent state. These cells were distributed into 60 mL Pyrex flasks (all glassware was sterilized (autoclaved 110 °C, 20 min) before use in the experiments) (Figure 9) and irradiated for 10 h by artificial light with an Atlas Suntest solar simulator under an irradiance of 500 W·m^−2^ in the wavelength range 280–700 nm. After irradiation, the cells were placed in darkness for 14 h and finally irradiated for 10 h. Exposure for 12 h at this intensity from the solar simulator corresponds to a natural daily dose measured at the surface of the sea in the north-western Mediterranean region in the summer [24]. The flasks were maintained at 17 °C by submersion in a water bath connected to a cryothermostat. One flask was also maintained in darkness at 17 °C as a control over the time course of the experiment. Bacteria control was performed on a bacterial community isolated from a culture of *E. huxleyi* [49]. The cells were transferred, after centrifugation (5000 rpm for 20 min), into 100 mL of filtered (0.2 μm) natural seawater, distributed in a 60 mL Pyrex flask and then irradiated as described above. To confirm that singlet oxygen is involved in the formation of hydroperoxide, senescent *E. huxleyi* cells were centrifuged (3500 rpm for 5 min), transferred into 5 mixtures of deuterium water (D_2_O, Sigma, 151882, St. Louis, MO, USA) and MQ water (from 0% to 100% of D_2_O, salinity adjusted to 37‰) and then irradiated for 4 h as described above.

To monitor the degradation of hydroperoxides, *E. huxleyi* cells were transferred to old seawater and the algal and bacterial growths were stopped by adding HgCl_2_ (final concentration 0.005 M). This poison was employed in order to avoid bacterial consumption of photoproducts in darkness. The dead non-axenic phytoplanktonic culture was subsequently transferred to a 250 mL Pyrex bottle covered with aluminium foil (Figure 9) and closed by a stopper equipped with a full sun spectral filter (λ > 280 nm). Irradiation of the contents of the flask was performed at 17 °C, using the solar simulator under an irradiance of 500 W·m^−2^. The contents of the flask were irradiated for 8.5 h and then incubated in darkness for 25 h to monitor the degradation of photochemically-produced hydroperoxides. One flask was also incubated in darkness at 17 °C during all the experiments as a control.

### 3.3. Lipid Analyses

After irradiation, a known volume of the irradiated culture was filtered on GF/F (Whatman, Maidstone, UK) filter for lipid analysis. Lipid biomarkers and their oxidation products were obtained after reduction with NaBH_4_ and subsequent saponification. All post-irradiation manipulations were conducted using foil-covered glassware to avoid photochemical artifacts. It is well known that metal ions can induce homolytic cleavage of hydroperoxides, and consequently promote free radical oxidation during procedures involving hot saponification [45]. The preliminary reduction of the hydroperoxides ensured that such free radical oxidation artifacts were avoided during the alkaline hydrolysis step. Due to the use of NaBH_4_-reduction during the treatment, it was not possible to distinguish hydroperoxides from their ketonic and alcoholic degradation products.

Note that *cis*-vaccenic and oleic acid oxidation products were obtained from the measurement of two groups of six isomeric hydroxyacids that resulted from the NaBH_4_ reduction of the corresponding hydroperoxyacids, *i.e.*, 11-hydroxyoctadec-*trans*-12-enoic, 12-hydroxyoctadec-*trans*-10-enoic, 13-hydroxyoctadec-*trans*-11-enoic, 13-hydroxyoctadec-*cis*-11-enoic, 10-hydroxyoctadec-*trans*-11-enoic, and 10-hydroxyoctadec-*cis*-11-enoic acids for *cis*-vaccenic acid (Figures 2 and 3) and 9-hydroxyoctadec-*trans*-10-enoic, 10-hydroxyoctadec-*trans*-8-enoic, 11-hydroxyoctadec-*trans*-9-enoic, 11-hydroxyoctadec-*cis*-9-enoic, 8-hydroxyoctadec-*trans*-9-enoic, and 8-hydroxyoctadec-*cis*-9-enoic acids for oleic acid [26].

**Figure 10:**
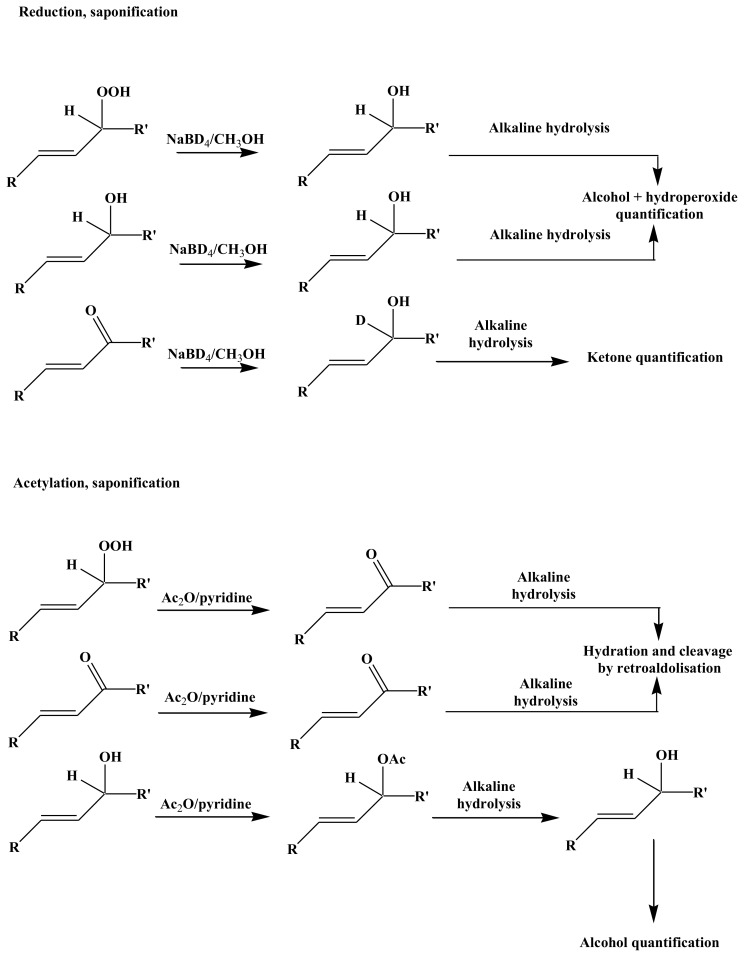
Sample chemical treatments for alcohol, ketone, and hydroperoxide quantification.

A different extraction method was employed to analyze the hydroperoxide degradation products (Figure 10). Samples (30 mL) were ultrasonically extracted (15 min) with chloroform: methanol: water (1:2:0.8, *v*/*v*/*v* [50]), then Milli-Q water was added to the extract to yield a final chloroform: methanol: water ratio of 1:2:2.3 (*v*/*v*/*v*) to initiate phase separation. Lipids were recovered in the lower chloroform phase, which was dried over anhydrous Na_2_SO_4_, filtered, and concentrated by rotary evaporation at 40 °C. The residue was then dissolved in 4 mL of dichloromethane and separated into two equal subsamples. Degradation products were obtained for the first subsample after acetylation and saponification, and they were obtained for the second one after reduction with NaBD_4_ and saponification (Figure 10).

#### 3.3.1. Reduction

The hydroperoxides were reduced to alcohols in methanol (25 mL) by adding excess NaBH_4_ or NaBD_4_ (10 mg per sample) using manual stirring (30 min at 20 °C) [51]. During this treatment, ketones are also reduced to their corresponding alcohols and the possibility of some ester cleavage cannot be totally excluded.

#### 3.3.2. Acetylation

The residues obtained after evaporation were taken up in 300 mL of a mixture of pyridine and acetic anhydride (2:1, *v*/*v*), allowed to react at 50 °C overnight, and then evaporated to dryness under nitrogen. Under these conditions, hydroperoxides are quantitatively transformed into their corresponding ketones [52].

#### 3.3.3. Alkaline Hydrolysis

Saponification was performed on the reduced samples and on the acetylated samples (taken in 25 mL of methanol) [26,27]. Twenty-five milliliters of water and 2.8 g of potassium hydroxide were added and the mixture was directly saponified by refluxing for 2 h. The aqueous phase was then acidifed with hydrochloric acid (pH 1) and subsequently extracted three times with dichloromethane. The combined dichloromethane extracts were dried over anhydrous Na_2_SO_4_, filtered, and concentrated by rotary evaporation at 40 °C to obtain the saponified fraction.

#### 3.3.4. Derivatization

The residues were taken up in 300 μL of a pyridine and bis(trimethylsilyl)trifluoroacetamide (BSTFA, Supelco, Bellefonte, PA, USA) mixture (2:1, *v*/*v*) and silylated at 50 °C for 1 h [53]. After evaporation to dryness under nitrogen, the residues were taken up in a suitable volume of a mixture (1:1, *v*/*v*) of ethyl acetate and BSTFA (to avoid desilylation of easily silylated compounds) and analyzed by gas chromatography-electron impact mass spectrometry (GC-EIMS).

### 3.4. Identification and Quantification of Lipid Biomarkers and Their Degradation Products by Gas Chromatography—Electron Impact Mass Spectrometry

The compounds were identified by comparing their retention times and mass spectra with those of standards (when available) and quantified (calibration with external standards) by GC-EIMS (Agilent 5975C mass spectrometer connected to a 6850 gas chromatograph, Santa Clara, CA, USA). For low concentrations, or in the case of co-elutions, quantification was achieved using selected ion monitoring (SIM). The following operating conditions were employed: 30 m × 0.25 mm (i.d.) fused silica capillary column coated with HP-5MS (Agilent; film thickness: 0.25 μm); oven temperature programmed from 70 to 130 °C at 20 °C·min^−1^, from 130 to 250 °C at 5 °C·min^−1^, and then from 250 to 300 °C at 3 °C·min^−1^; carrier gas (He), 1.0 bar; injector (pulsed splitless) temperature, 250 °C; electron energy, 70 eV; source temperature, 230 °C; and cycle time, 0.2 s. Standard products from the oxidation of oleic and vaccenic acids were obtained according to previously described procedures [26]. The products resulting from the degradation of the epoxy acids during the treatment (chlorohydrins, methoxyhydrins and diols) were quantified using a standard of 9,10-dihydroxyoctadecanoic acid produced after oxidation of oleic acid with OsO_4_.

### 3.5. Chlorophyll a Analyses

Five milliliters of samples were filtered through GF/F filters (previously cleaned by refluxing in CH_2_Cl_2_/MeOH 2:1 overnight) and then the filters were transferred into a glass tube and stored at −20 °C. After adding 5 mL of pure methanol (Fisher Scientific, Loughborough, UK), the tube was closed and the extraction was performed in darkness at 4 °C for 30 min. The sample fluorescence was directly measured with a Turner Designs fluorimeter equipped with a F4T5 Blue lamp, a 5–60 (450 nm) excitation filter and a 2–64 (660 nm) high-pass emission filter. The fluorimeter was calibrated with pure chlorophyll *a* (Sigma C5753, St. Louis, MO, USA) dissolved in methanol (96%). The concentration of this solution was determined by spectrophotometry using the specific absorption coefficient of 77 L g^−1^·cm^−1^ at 663 nm [54]. Correcting for phaeopigment interferences was performed using the acidification method [55], which required a second fluorescence measurement after adding of 50 μL of hydrochloric acid (0.5 N) to the extract.

## 4. Conclusions

Photodegradation processes were investigated in non-axenic senescent cultures of the haptophyte *E. huxleyi*. Specific attention was devoted to the transfer of singlet oxygen from the phytodetritus to the attached bacteria. Oleic acid (component of phytoplanktonic and bacterial cells) and *cis*-vaccenic acid (specific component of Gram-negative bacteria) were employed to compare the effects of the photodegradation processes in these two types of organisms.

The first experiment confirmed some previous results concerning the transfer of singlet oxygen from senescent phytoplanktonic cells to attached bacteria [12,21,51] and revealed a significant correlation between the concentration of degraded chlorophyll *a* (sensitizer) in the phytodetritus, and the photodegradation state of the attached bacteria (concentration of *cis*-vaccenic photoproducts).

The behavior of the photochemically-produced hydroperoxyacids in bacteria and phytoplanktonic cells was investigated during PAR + UV irradiation and in darkness. In both organisms, homolytic cleavage of hydroperoxides induced by UV irradiations and/or metal ions, resulted in the production of similar proportions of ketoacids and hydroxyacids. In darkness, the additional involvement of peroxygenases (enzymes catalyzing the transfer of an oxygen atom from the hydroperoxide group of hydroperoxyacids to the double bonds of unsaturated fatty acids) [44] resulted in the simultaneous production of hydroxyacids and epoxyacids. These enzymes play an important protective role against the deleterious effects of fatty acid hydroperoxides in bacteria and phytoplankton [44]. Indeed, organic hydroperoxides can initiate lipid peroxidation chain reactions leading to DNA and membrane damage [56]; their elimination is therefore particularly important for living cells.

It is generally accepted that marine bacteria colonize phytoplankton-derived particles, and significantly contribute to the degradation of these aggregates during their sedimentation. In this paper, we could demonstrate that phytodetritus can also participate in the degradation of attached bacteria during their stay within the euphotic layer. Singlet oxygen transfer from the phytodetritus to attached heterotrophic bacteria could induce strong oxidative damage in these organisms (not only on unsaturated fatty acids but also on proteins and nucleic acids [57,58]), which has the potential to limit their growth and therefore contribute to a better preservation of algal organic matter during the sedimentation. Note that the surprising recalcitrance of phytodetritus towards biodegradation processes observed during the Arctic midnight sun period was recently attributed to the strong photodegradation state of heterotrophic bacteria associated with this material [33], which likely resulted from the efficient transfer of singlet oxygen from the photodegraded phytoplanktonic cells to the attached bacteria.

## Figures and Tables

**Table 1:** Sum of the *cis*-vaccenic acid concentration and the oxidation products concentration during the first irradiation of senescent culture of a non-axenic culture of *E. huxleyi.*

*Cis-*vaccenic acid concentration (μg·L^−1^)	*Cis-*vaccenic acid oxidation products concentration (μg·L^−1^)	Sum (μg·L^−1^)
308.7	18.0	326.7
292.7	40.0	323.7
260.8	38.2	299.0
258.7	69.8	328.6
257.9	63.4	321.2
		Average: 321.6 ± 13.3

**Table 2:** Reaction rate constants (*k*) of *cis*-vaccenic photoproducts formation (h^−1^) observed during irradiation of senescent culture of different non-axenic strains of *E. huxleyi.*

*E. huxleyi* strain	Reaction rate constants (k) of *cis*-vaccenic photoproducts formation (h^−1^)
TW1	0.152
CS814	0.138
CS57	0.173
	Average : 0.153 ± 0.018

**Table 3:** Increase of *cis*-vaccenic acid photooxidation products (relative to the pure H_2_O sample) observed after irradiation of non-axenic senescent *E. huxleyi* cells in different mixtures of D_2_O and H_2_O.

Percentage of D_2_O	Increase of *cis*-vaccenic photooxidation products relative to the pure H_2_O sample (%)
0	0
25	2.5
50	76.5
75	50.2
100	70.4

**Table 4:** Percentage of *cis*-vaccenic acid photooxidation products relative to the residual parent compound, observed after irradiation of non-axenic *E. huxleyi* cells and isolated bacterial community.

Irradiation time (h)	Non-axenic senescent cells of *E. huxleyi*	Bacterial community isolated from *E. huxleyi* cells
0	5.8	0
7.5	27.0	0
31.5	9.9	0.6

## References

[1] Cho B.C., Azam F. (1988). Major role of bacteria in biogeochemical fluxes in the ocean’s interior. Nature.

[2] Eppley R., Peterson B.J. (1979). Particulate organic matter flux and planktonic new production in the deep ocean. Nature.

[3] Boyd P.W., Trull T.W. (2007). Understanding the export of biogenic particles in oceanic waters: Is there consensus?. Progress Oceanogr.

[4] Thunell R., Benitez-Nelson C., Varela R., Astor Y., Muller-Karger F. (2007). Particulate organic carbon fluxes along upwelling-dominated continental margins: Rates and mechanisms. Glob. Biogeochem. Cycles.

[5] Turley C., Mackie P. (1994). Biogeochemical significance of attached and free-living bacteria and the flux of particles in the NE Atlantic Ocean. Mar. Ecol. Progress Ser.

[6] Sempéré R., Yoro S.C., van Wambeke F., Charrière B. (2000). Microbial decomposition of large organic particles in the northwestern Mediterranean Sea: An experimental approach. Mar. Ecol. Progress Ser.

[7] Goutx M., Wakeham S.G., Lee C., Duflos M. (2007). Composition and degradation of marine particles with different settling velocities in the northwestern Mediterranean Sea. Limnol. Oceanogr.

[8] Tamburini C., Goutx M., Guigue C., Garel M., Lefèvre D., Charrière B., Sempéré R., Pepa S., Peterson M.L., Wakeham S.G., Lee C. (2009). Effects of hydrostatic pressure on microbial alteration of sinking fecal pellets. Deep Sea Res. Part II.

[9] Rontani J.-F., Zabeti N., Wakeham S.G. (2011). Degradation of particulate organic matter in the equatorial Pacific Ocean: Biotic or abiotic?. Limnol. Oceanogr.

[10] Mayer L.M., Schick L.L., Bianchi T.S., Wysocki L.A. (2009). Photochemical changes in chemical markers of sedimentary organic matter source and age. Mar. Chem.

[11] Rontani J.-F. (2001). Visible light-dependent degradation of lipidic phytoplanktonic components during senescence: A review. Phytochemistry.

[12] Christodoulou S., Joux F., Marty J.-C., Sempéré R., Rontani J.-F. (2010). Comparative study of UV and visible light induced degradation of lipids in non-axenic senescent cells of Emiliania huxleyi. Mar. Chem.

[13] Foote C, Pryor W.A. (1976). Photosensitized Oxidation and Singlet Oxygen: Consequences in Biological Systems.

[14] Knox J.P., Dodge A.D. (1985). Singlet oxygen and plants. Phytochemistry.

[15] Halliwell B. (1987). Oxidative damage, lipid peroxidation and antioxidant protection in chloroplasts. Chem. Phys. Lipids.

[16] Nelson J.R. (1993). Rates and possible mechanism of light-dependent degradation of pigments in detritus derived from phytoplankton. J. Mar. Res.

[17] Merzlyak M.N., Hendry G.A.F. (1994). Free radical metabolism, pigment degradation and lipid peroxidation in leaves during senescence. Proc. R. Soc. Edinb.

[18] Suwa K., Kimura T., Schaap A.P. (1977). Reactivity of singlet molecular oxygen with cholesterol in a phospholipid membrane matrix. A model for oxidative damage of membranes. Biochem. Biophys. Res. Commun.

[19] Rontani J.-F., Matsumoto T. (2008). Photooxidative and Autoxidative Degradation of Lipid Components during the Senescence of Phototrophic Organisms.

[20] Rontani J.-F., Rabourdin A., Pinot F., Kandel S., Aubert C. (2005). Visible light-induced oxidation of unsaturated components of cutins: A significant process during the senescence of higher plants. Phytochemistry.

[21] Rontani J.-F., Koblízek M., Beker B., Bonin P., Kolber Z.S. (2003). On the origin of *cis*-vaccenic acid photodegradation products in the marine environment. Lipids.

[22] Sicre M., Paillasseur J., Marty J.-C., Saliot A. (1988). Characterization of seawater samples using chemometric methods applied to biomarker fatty acids. Org. Geochem.

[23] Keweloh H., Heipieper H.J. (1996). *Trans* unsaturated fatty acids in bacteria. Lipids.

[24] Abboudi M., Surget S.M., Rontani J.-F., Sempéré R., Joux F. (2008). Physiological alteration of the marine bacterium Vibrio angustum S14 exposed to simulated sunlight during growth. Curr. Microbiol.

[25] Garcia-Pichel F. (1994). A model for internal self-shading in planktonic organisms and its implications for the usefulness of ultraviolet sunscreens. Limnol. Oceanogr.

[26] Marchand D., Rontani J.-F. (2001). Characterisation of photo-oxidation and autoxidation products of phytoplanktonic monounsaturated fatty acids in marine particulate matter and recent sediments. Org. Geochem.

[27] Marchand D., Marty J.-C., Miquel J.-C., Rontani J.-F. (2005). Lipids and their oxidation products as biomarkers for carbon cycling in the northwestern Mediterranean Sea: Results from a sediment trap study. Mar. Chem.

[28] Christodoulou S., Marty J.-C., Miquel J.-C., Volkman J.K., Rontani J.-F. (2009). Use of lipids and their degradation products as biomarkers for carbon cycling in the northwestern Mediterranean Sea. Mar. Chem.

[29] Minnock A., Vernon D., Schofield J. (1996). Photoinactivation of bacteria. Use of a cationic water-soluble zinc phthalocyanine to photoinactivate both gram-negative and gram-positive bacteria. J. Photochem. Photobiol. B.

[30] Merchat M., Bertolini G., Giacomini P., Villanueva A, Jori G. (1996). Meso-substituted cationic porphyrins as efficient photosensitizers of gram-positive and gram-negative bacteria. J. Photochem. Photobiol. B.

[31] Merchat M., Spikes J.D., Bertoloni G., Jori G. (1996). Studies on the mechanism of bacteria photosensitization by meso-substituted cationic porphyrins. J. Photochem. Photobiol. B.

[32] Zanin I.C.J., Gonçalves R.B., Junior A.B., Keith Hope C., Pratten J. (2005). Susceptibility of Streptococcus mutans biofilms to photodynamic therapy: An *in vitro* study. J. Antimicrob. Chemother.

[33] Rontani J.-F., Charriere B., Forest A., Heussner S., Vaultier F., Petit M., Delsaut N., Fortier L., Sempéré R. (2012). Intense photooxidative degradation of planktonic and bacterial lipids in sinking particles collected with sediment traps across the Canadian Beaufort Shelf (Arctic Ocean). Biogeosciences.

[34] Frimer A.A. (1979). The reaction of singlet oxygen with olefins: The question of mechanism. Chem. Rev.

[35] Frankel E.N. (1998). Lipid Oxidation.

[36] Porter N.A., Caldwell S.E., Mills K.A. (1995). Mechanisms of free radical oxidation of unsaturated lipids. Lipids.

[37] Aebisher D., Azar N.S., Zamadar M., Gandra N., Gafney H.D., Gao R., Greer A. (2008). Singlet oxygen chemistry in water: A porous vycor glass-supported photosensitizer. J. Phys. Chem. B.

[38] Jin N., Lahaye D.E., Groves J.T. (2010). A “push-pull” mechanism for heterolytic O–O bond cleavage in hydroperoxo manganese porphyrins. Inorg. Chem.

[39] Dahl T.A., Midden W.R., Hartman P.E. (1989). Comparison of killing of gram-negative and gram-positive bacteria by pure singlet oxygen. J. Bacteriol.

[40] Horspool W.M., Armesto D, Horwood E. (1992). Organic Photochemistry: A Comprehensive Treatment.

[41] Schaich K.M. (2005). Lipid Oxidation: Theoretical Aspects. Bailey’s Industrial Oil and Fat Products.

[42] Berti G. (1973). Stereochemical aspects of the synthesis of 1,2-epoxides. Top. Stereochem.

[43] Fossey J., Lefort D., Sorba J, Masson (1995). Free Radicals in Organic Chemistry.

[44] Blée E., Schuber F. (1990). Efficient epoxidation of unsaturated fatty acids by a hydroperoxide-dependent oxygenase. J. Biol. Chem.

[45] Pokorny J, Chan H.W. (1987). Major Factors Affecting the Autoxidation of Lipids.

[46] Schaich K.M. (1992). Metals and lipid oxidation. Contemporary issues. Lipids.

[47] Testa A.C. (1964). Photosensitized *cis*-*trans* isomerization of methyl oleate. J. Org. Chem.

[48] Jacobson F.S., Morgan R.W., Christmanlf M.F., Amesll B.N. (1989). An alkyl hydroperoxide reductase from *Salmonella typhimurium* involved in the defense of DNA against oxidative damage. J. Biol. Chem.

[49] Rontani J.-F., Harji R., Guasco S., Prahl F.G., Volkman J.K., Bhosle N.B., Bonin P. (2008). Degradation of alkenones by aerobic heterotrophic bacteria: Selective or not?. Org. Geochem.

[50] Volkman J.K., Farmer C.L., Barrett S.M., Sikes E.L. (1997). Unusual dihydroxysterols as chemotaxonomic markers for microalgae from the order pavlovales (haptophyceae). J. Phycol.

[51] Marchand D., Rontani J.-F. (2003). Visible light-induced oxidation of lipid components of purple sulfur bacteria: A significant process in microbial mats. Org. Geochem.

[52] Mihara S., Tateba H. (1986). Photosensitized oxygenation reactions of phytol and its derivatives. J. Org. Chem.

[53] Pierce A.E. (1982). Silylation of Organic Compounds.

[54] Marker A.F.H. (1972). The use of acetone and methanol in the estimation of chlorophyll in the presence of phaeophytin. Freshw. Biol.

[55] Holm-Hansen O., Lorenzen C.J., Holmes R.W., Strickland J.D.H. (1965). Fluorometric determination of chlorophyll. ICES J. Mar. Sci.

[56] Halliwell B., Gutteridget J.M.C. (1984). Oxygen toxicity, oxygen radicals, transition metals and disease. Biochem. J.

[57] Dias Cavalcante A.K., Martinez G.R., di Mascio P., Martins Menck C.F., Lucymara Fassarella A.-L. (2002). Cytotoxicity and mutagenesis induced by singlet oxygen in wild type and DNA repair deficient Escherichia coli strains. DNA Repair.

[58] Davies M.J. (2005). The oxidative environment and protein damage. Biochim. Biophys. Acta.

